# Web-based analysis of the mouse transcriptome using Genevestigator

**DOI:** 10.1186/1471-2105-7-311

**Published:** 2006-06-21

**Authors:** Oliver Laule, Matthias Hirsch-Hoffmann, Tomas Hruz, Wilhelm Gruissem, Philip Zimmermann

**Affiliations:** 1 Institute of Plant Sciences, ETH Zurich, 8092 Zurich, Switzerland; 2 Institute of Theoretical Computer Science, ETH Zurich, 8092 Zurich, Switzerland

## Abstract

**Background:**

Gene function analysis often requires a complex and laborious sequence of laboratory and computer-based experiments. Choosing an effective experimental design generally results from hypotheses derived from prior knowledge or experimentation. Knowledge obtained from meta-analyzing compendia of expression data with annotation libraries can provide significant clues in understanding gene and network function, resulting in better hypotheses that can be tested in the laboratory.

**Description:**

Genevestigator is a microarray database and analysis system allowing context-driven queries. Simple but powerful tools allow biologists with little computational background to retrieve information about when, where and how genes are expressed. We manually curated and quality-controlled 3110 mouse Affymetrix arrays from public repositories. Data queries can be run against an annotation library comprising 160 anatomy categories, 12 developmental stage groups, 80 stimuli, and 182 genetic backgrounds or modifications. The quality of results obtained through Genevestigator is illustrated by a number of biological scenarios that are substantiated by other types of experimentation in the literature.

**Conclusion:**

The Genevestigator-Mouse database effectively provides biologically meaningful results and can be accessed at .

## Background

The development of functional genomics technologies has led in recent years to a proliferation of databases for storage and delivery of microarray data. Since the introduction of the MIAME standard [1] and associated community- level annotation guidelines [2-4], experimental descriptions have become more precise, allowing a better understanding and reproducibility of experiments, as well as more efficient querying possibilities. Several databases offer tools to browse, query and download experiments. However, in most cases, data is provided as is, without removal of biased data after systematic processing with quality-control measures. Furthermore, the focus of most microarray databases has been in storage and retrieval of experiments, but only few provide analysis tools optimally interacting with the database. In parallel to these developments, several web-based tools have recently been developed specifically for the analysis of individual microarray experiments, such as RACE [5] or ArrayQuest [6]. High-throughput technologies allow to streamline the same type of analysis for large numbers of genes or proteins. A major challenge for scientists in this respect is the sparsity of the data sets, i.e. the low number of measurements relative to the immense number of simultaneously tested elements. The analysis of such data structures often cannot make use of many classical statistical procedures and calls for the development of novel statistical approaches, such as sparse graphical modeling [7] or computational approaches that allow to compile result summaries combining data and annotations.

Genevestigator [8] is a high-quality database combined with tools to create such result summaries. It reveals novel and diverse information about when, where and how genes are expressed in order to foment both discovery and hypothesis generation. In fact, hypothesis-driven biological research solicits models to represent biological processes. Once models are created, they are tested against experimental results. The design of models and of the proper experiments allowing to effectively conclude about their validity is a crucial step in the discovery process. The availability, diversity, robustness, and correct interpretation of prior experimental results, such as those from microarray experiments, are therefore instrumental in formulating new hypotheses and models, as well as in designing the proper experiments to test them. Genevestigator-Mouse aims at providing easy-to-use but powerful tools that enable biologists to obtain context-driven information about the expression of the mouse transcriptome. The information obtained helps to validate existing hypotheses, as well as to formulate new hypotheses or to design novel experiments.

## Construction and content

### Data source, processing, and annotation

Data was downloaded via FTP from public repositories such as Gene Expression Omnibus [9], ArrayExpress [10], MUSC [11], PEPR [12], ChipperDB [13] or NIH Neuroscience Microarray Consortium [14]. Raw data (CEL files) were normalized with the Affy package from Bioconductor [18] using the MAS5 algorithm. Experiment annotations were retrieved from public repositories, from original publications, and occasionally directly from the authors. Anatomy ontologies, of which 160 are currently represented in the database, are based on definitions provided by the Edinburgh Mouse Atlas Project and available at Mouse Genome Informatics [15]. Developmental stages are partitioned into 27 pre-natal [16] and 5 post-natal stages. In the latter case, stages were defined based on a log(4) scale of time units (days) after birth. Genetic modifications were systematically annotated according to the underlying mutagenesis methods, e.g. targeted deletion or ENU mutagenesis and including, if possible, information about which genes were affected. As for treatments and stimuli, several major categories currently cover 80 treated samples (+) and their corresponding controls (-). Data for the mapping of probe sets to gene identifiers were obtained from the Affymetrix website [17]. Currently, either probe set or UniGene identifiers can be used for querying the database.

### Quality control

A prerequisite for the type of analysis provided by Genevestigator is data comparability between experiments. Although methods how to combine data from different technological platforms and laboratories are still a matter of debate, the common analysis of data from a single organism, a single platform such as the Affymetrix system, and a single array type has so far proven to successfully reveal biological mechanisms. In fact, as can be verified from recent publications, results obtained from Genevestigator with Arabidopsis could be substantiated by other experimental techniques such as RT-PCR, reporter gene analysis, mutant phenotyping, or other microarray studies. In order to maximize comparability, quality-control (QC) measures were applied to raw and normalized data. The current QC protocol uses several Bioconductor [18] packages (simpleaffy, AffyQCReport, AffyPLM) and in-house R code. QC results include signal intensity box-plots and density-plots, Actin and GAPDH QC statistics, RLE, NUSE, RNA digestion plots, positive and negative border element plots, as well a correlation matrix. QC reports are publicly available. Array data containing flaws or biases, or of which RNA was strongly degraded, are flagged and excluded by default from the data analysis tools. Currently, approximately 3.4% of arrays did not fulfil the QC criteria. However, users can decide whether to use all the available data by inactivating the default "Quality Control" option.

### Application

The software was developed as a PHP/MySQL application running on a Linux Apache Web server. Javascript and cookies must be activated for browsers to correctly run the application. More details about methods and algorithms used can be found in the documentation section on the Genevestigator website [19].

### Database and analysis tools

The database contains both 12 K and 40 K Affymetrix arrays (MG-U74Av2 and Mouse430 2.0). As of June 2006, 3110 arrays from 166 experiments were curated, quality-controlled and annotated according to controlled vocabularies. Two types of queries can be run: gene-centric (how is gene X expressed in a series of conditions?) or genome-centric (which genes are expressed in a pre-selection of conditions?). The tools described below contain either one or both types. Data can be viewed either in linear or log(2)-scale relative to the following categories:

*Experiments and arrays *(Digital Northern): the digital Northern visualizes signal intensity values and present/absent calls of a subset of genes across a selection of experiments.

*Anatomy *(Gene Atlas): this tool reveals the anatomy-specific profiles of single query genes, or reversely allows to identify genes expressed specifically in a selection of organs/tissues.

*Stimulus *(Response Viewer): this tool shows the responses of genes to a compendium of stimuli. Results can be sorted such as to rapidly identify those factors most affecting the expression of query genes. Reversely, it allows to find genes expressed specifically in a subset of conditions.

*Development *(Gene Chronologer): changes in gene expression throughout the life cycle of the mouse, which is grouped into 7 embryo and 5 post-natal stage groups, can be visualized. Reversely, one can search for genes expressed specifically at given stages of development.

*Mutation *(Mutant Surveyor): this tool is similar to the Response Viewer, plotting the responses of a query gene to a number of genetic backgrounds or modifications (e.g. gene knock-out or overexpression).

*Multiple genes *(Meta-Analyzer): expression profile summaries of a larger number of genes with respect to anatomy, development, or stimulus can be queried.

*Documentation: *the documentation and FAQ sections provide important technical and practical information about the tools, such as statistical procedures, probe set specificity, normalization, how to interpret data, and precautions to avoid over-interpretation.

*Database: *this page provides information about all experiments stored in our database, including data source repositories, authors, publications, dates, original file names, Genevestigator file names, annotations, quality-control results, and links to external information. Since Genevestigator is basically a data analysis tool and not a repository, we do not provide data bulk download options and therefore recommend users to download the data from the original repositories.

## Utility and discussion

A validation and discovery study is presented to illustrate some of the querying possibilities and to assess the quality of the output by comparison to prior biological knowledge obtained from the literature. Besides confirming previously known mechanisms, we present novel findings and hypotheses about the regulation of given genes in mouse.

Our database encompasses a wide range of experimentally annotated gene expression data covering diverse categories i.e. different tissues, developmental stages, treatments and genetic modifications. Since the averages computed for each category are based on data from a variety of experimental setups that are not necessarily systematic throughout all categories, care must be taken not to over-interpret results. Our general hypothesis is that an increase in the number of experiments and replicates per defined category generally attenuates experiment specific effects in favor of global trends. To verify whether results from Genevestigator show robust and reproducible trends, we selected from the literature a number of genes with expression profiles specific to organs or to developmental stages, or genes responsive to certain stimuli or to genetic modifications and analyzed their respective expression patterns generated by Genevestigator.

**Figure 1:**
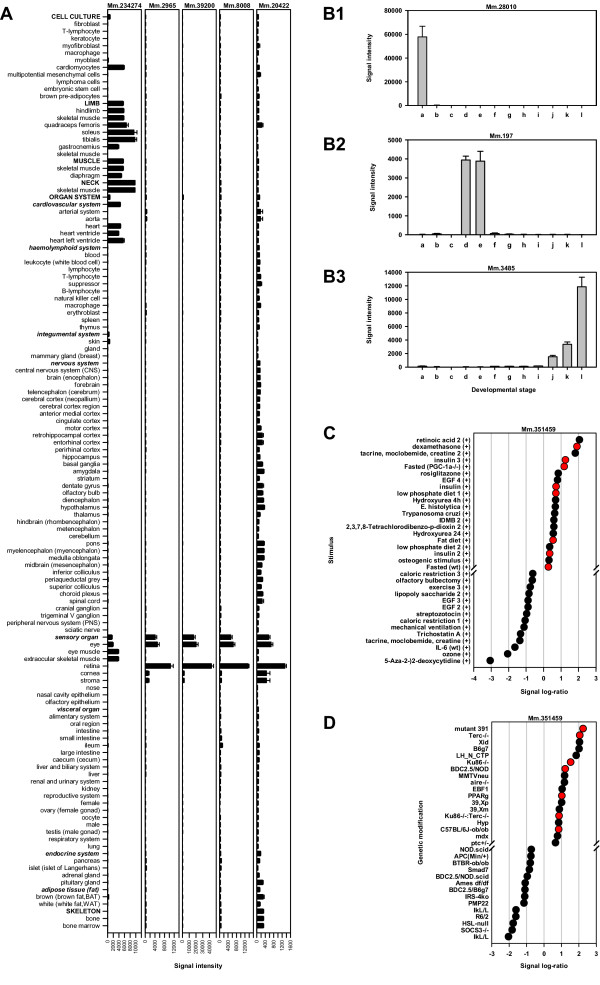
**Validation of expression profiles**. The results shown were calculated from 2138 arrays of type MG-U74Av2 (12 K). Similar results can be obtained using arrays of type Mouse430 2.0 (40 K) for categories available in both array types. For each gene, the signal intensity value indicated in each category is the average signal intensity value (of the corresponding probe set) from all arrays within this category. Average signal intensity values are available throughout all shown categories for all probe sets represented on the 12 K array. A. Anatomy expression profiles of genes shown in the literature to be preferentially expressed in muscle and heart (Mm.234274) or in retina (Mm.2965, Mm.39200, Mm.8008, Mm.20422). B1-B3. Development expression profiles of three genes previously shown to be expressed in oocytes and up to the 8-cell stage (Mm.28010), between E7.5 and E8.5 (Mm.197), and in adult mouse but low in embryo or newborn mice (Mm.3485). Developmental stages are defined as pre-natal (given in Theiler stages (TS); a: TS-1 to 2, b: TS-3 to 5, c: TS-6 to 9, d: TS-10 to 13, e: TS-14 to 18, f: TS-19 to 23, g: TS-24 to 27) or post-natal (given in days after birth; h: 0–3, i: 4–15, j: 16–63, k: 64–255, l: > 256). C and D. Response of Sirt1 (Mm.351459) to a set of stimuli (C) and to genetic modifications (D). Only treatments or mutations inducing strong up or down regulation are shown. Effects described in the text are highlighted. These precompiled responses are log-ratios from the average signal intensity values of treatment versus control samples from a variety of experiments and tissues.

First, using the Anatomy profiles (Gene Atlas tool), four genes which have a well-documented retina specific expression (CRX [Mm. 8008] [20], Rho [Mm.2965], PDE6 [Mm.39200] [21], and Nrl [Mm.20422] [22]), in fact showed a strong expression in the retina (and its parent categories), but no or only weak signals in other organs (Fig. 1A). Furthermore, the spatial expression of both Titin [Mm.26579], known to play a critical role for both heart [23] and muscle [24] and BOP (Smyd1 [Mm.234274]), a heart and muscle specific transcription factor [25] was restricted to the corresponding tissues (Fig. 1A) [see Additional file 1].

Second, to verify the reliability of the Development profiles (Gene Chronologer tool), we looked for genes annotated as being developmentally regulated. Only a handful of early embryonic genes have been described to date. Among those we tested the RING finger protein gene Rnf33 [Mm.28010] and Hoxal [Mm.197] a homeobox transcription factor that regulates embryonic patterning and organogenesis. Transcription of Rnf33 has been shown to occur already in the mouse oocyte but not beyond the eight-cell stage nor in adult tissues [26]. Hoxal expression starts at E7.5 and begins to retreat caudally by day E8.5 [27]. Both genes were found to be expressed solely in the corresponding embryonic developmental stage groups (Fig. 1B1 and 1B2) [see Additional file 1]. In contrast, the gene encoding hemopexin (hx, [Mm.3485]), a plasma glycoprotein known to be only lowly expressed in embryos and newborn mice [28] showed an adult stage specific expression profile (Fig. 1B3) with the strongest signal at the latest stage, which reflects the fact that hx gene expression reaches adult level not until the first year of age [28].

Third, to test the Genevestigator output in terms of responses to different stimuli (Response Viewer) and to genetic modifications (Mutant Surveyor), we used Sirt1 [Mm.351459], a nuclear deacetylase that is closely associated with the longevity elicited by caloric restriction (CR, [29], Fig. 1C) [see Additional file 1]. In mammals a characteristic set of physiological changes takes place during CR. Among those changes is the use of dietary fat or fat mobilized from white adipose tissue for energy, and a large reduction in blood insulin levels accompanied by an increase in insulin sensitivity. It was shown that the flux of metabolites under fasting conditions leads to an increase in Sirt1 mRNA levels and that Sirt1 also controls glucose metabolism through the regulation of PPAR co-activator 1 [30]. The Response Viewer correctly showed an up regulation of Sirt1 in fasted mice, in mice which were put on a fat diet, as well as in response to several insulin treatments. No change or even a slight down regulation in Sirt1 expression was observed in caloric restriction treatments performed with Ames mice. This is consistent with the fact that the pathways responsible for extending lifespan in the dwarfs and in CR animals are not identical [31]. In addition, we found Sirt1 severely up regulated in response to dexamethasone, in accordance with the reported increase of Sirt1 protein levels upon dexamethasone treatment measured by western blot analysis of 3T3-L1 fibroblasts [32]. The Mutant Surveyor analysis revealed a substantial increase of Sirt1 mRNA levels in mice with a truncated growth hormone receptor (mutant 391), which are characterized by marked male obesity associated with hyperglycemia; in BDC2.5/NOD mice, which develop a mild cellular infiltrate in the pancreatic islets of Langerhan's (insulitis); and in C57BL/6J-ob/ob mice, which exhibit a diabetes-like syndrome of hyperglycemia, glucose intolerance and elevated plasma insulin levels [33] (Fig. 1D). Hence, the Sirt1 expression profile in these mouse strains reflects its role in controlling glucose metabolism. Moreover, Sirt1 was found to be up regulated in PPAR over-expressing mice, which is consistent with the model that Sirt1 functions as a represser of genes that drive white adipocyte differentiation and fat storage [32]. Remarkably, among the genetically modified strains that showed increased Sirt1 expression were also telomerase-deficient mice (Terc-/-, [34]), mice defective in the Ku86 DNA repair protein (Ku86-/-, [35]) and mice double deficient for telomerase and Ku86 (Terc-/-/Ku86-/-, [36]) all characterized by a premature-aging phenotype. This in turn is consistent with the observation that the yeast Sirt1 ortholog Sir2, a central player in yeast aging, represses transcription near telomeres [37] and that both Sir2 and Ku86 are involved in non-homologous end joining, which is used to repair breaks in DNA by ligation of the free ends [38].

This validation approach clearly shows that Genevestigator is capable to detect biologically relevant trends in the expression profiles of individual genes by combining numerous normalized expression data sets using the same technical platform, i.e. the Affymetrix system.

**Figure 2:**
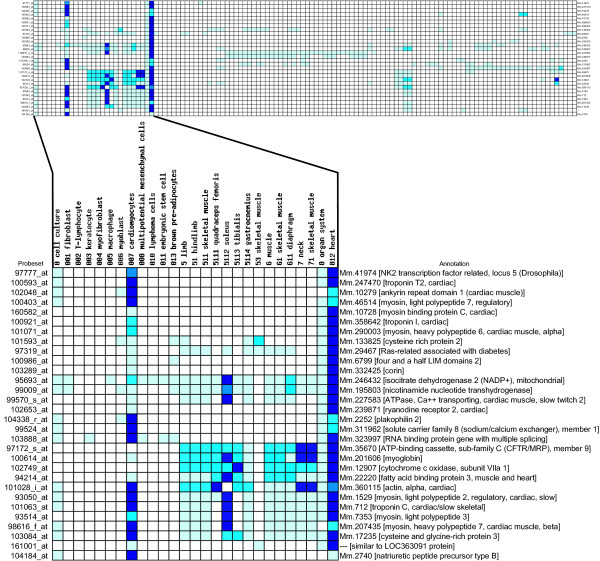
**Identification of marker genes**. Hierarchical cluster of the top 30 genes preferentially or specifically expressed in the heart. Results were calculated from 2138 arrays of type MG-U74Av2 (12 K).

We further performed a second approach to test whether Genevestigator can identify genes with known expression profiles. Using the anatomy genome-centric querying options, we created a list of the top 30 genes preferentially expressed in the heart (Figure 2) [see Additional file 1]. Of these, 12 could be spatially associated with the heart based on the annotation "cardiac". Further the list comprises the NK2 transcription factor related locus 5 (Nkx2-5, [Mm.26579]), a homeobox-containing gene specifically expressed in the developing heart [39]; the gene encoding corin [Mm.332425], a multiple-domain type II transmembrane serine protease highly expressed in the heart with a binding site for NKX2.5 transcription factors [40]; the cardiac troponin I (Tnni3, [Mm.358642]), a sarcomeric protein gene exclusively expressed in the cardiac muscle [41]; and FHL2 [Mm.6799], a LIM domain protein preferentially expressed in the heart [42]. The remaining genes are not yet associated with the heart or unknown, evincing the potential of Genevestigator to identify novel genes related to particular organs.

Based on our validation study, we conclude that with the current set of data, Genevestigator generates high quality results. Moreover, we expect that this quality will continue to rise as the size of the dataset increases.

## Conclusion

Using standardized protocols and systematic annotation, biologically relevant trends in gene expression can be identified. The combined average of several measurements within an annotation category increases the confidence about signal values obtained from these categories. At the same time, the quality and power of the tools will increase as more numerous and more diverse data become available and are included. Unfortunately, several interesting published experiments could not be included owing to lacking experiment annotation, highlighting the importance of proper and sufficient annotation with controlled vocabularies and following detailed guidelines. Furthermore, the availability of raw data (CEL files) generated with the 40 K array were sparsely available in repositories. We therefore encourage authors to provide also raw data when submitting their experiments to public repositories.

Due to the compilation of data from different labs, marker identification methods may be sensitive to arrays with signals resulting from very weak hybridization or signals that are in the saturation range. For this reason, applying strict quality-control measures based on raw data analysis is imperative for robust analysis. Genevestigator can be used either as a validation tool for genes already under study, as a tool to create new hypotheses, or to identify marker genes. Our results demonstrate that Genevestigator effectively identifies biological mechanisms and meta-data related marker genes.

## Availability and requirements

Project name: Genevestigator; Project homepage: ; Operating system: platform independent; Programming language: PHP4/MySQL; Licence: free access to current tools for academics; Any restrictions to use by non-academics: licence needed.

## Authors' contributions

OL, MHH and PZ performed software development, data curation, quality control and validation. PZ, TH and WG managed the project and contributed technically and intellectually. All authors participated in reading, approving and revising the manuscript.

## Supplementary Material

Additional File 1 Raw data for figures 1 and 2 were exported from the Genevestigator website [19]. Signal intensity values were obtained by normalization with MAS5 with a target value (TGT) of 1000. Click here for file
